# Use of Video-Assisted Thoracoscopic Surgery (VATS) in the Treatment of Primary Spontaneous Pneumothorax (PSP) in Children

**DOI:** 10.7759/cureus.42624

**Published:** 2023-07-28

**Authors:** Ceyhan Şahin, Hayriye Nihan Karaman Ayyıldız, Semih Mirapoğlu, Fatma Tuğba Güvenç, Zeliha Akis Yıldız, Mehmet Arpacik, Aytekin Kaymakçı, Zekeriya Ilce

**Affiliations:** 1 Pediatric Surgery, University of Health Sciences, Umraniye Training and Research Hospital, Istanbul, TUR

**Keywords:** chest tube, pneumothorax, spontan pneumothorax, thoracostomy tube, vats video assisted thoracoscopic surgery

## Abstract

Introduction: This study aims to review the primary spontaneous pneumothorax (PSP) patients we have treated and to discuss the results in terms of PSP treatment management and video-assisted thoracoscopic surgery (VATS) use in the light of the literature.

Methods: The study was designed retrospectively and conducted at a tertiary referral university hospital between January 1, 2015 and May 1, 2021. Patients under the age of 18 years with a diagnosis of pneumothorax (PTX) were included in the study. Medical records were analyzed in terms of clinical characteristics, demographic data, findings from imaging data, procedures performed, and course of the disease at hospital. Patients with no evidence of PTX on radiologic imaging (direct postero-anterior chest X-ray (PACXR) or thoracic computed tomography (TCT)), incomplete medical records for follow-up, history of trauma, and neonatal PTX were excluded from the study.

Results: The study was conducted on a total of 98 PTX cases in 69 patients, 61 (88.4%) males and eight (11.6%) females. The ages of the patients ranged between 13 and 17 years with a mean of 16.59 ± 0.95 years. While 48 (49%) PTX cases were treated with tube thoracostomy, 19 (19.4%) were treated with medical follow-up (nonsurgical treatment) and 31 (31.6%) were treated with VATS. A total of 31 VATS procedures were performed on 28 patients. The follow-up period after VATS ranged from tthree to 78 months, with a mean of 31.5 ± 20.3 months and a median of 28 months.

Conclusion: Our retrospective study showed that TCT scanning did not provide additional benefit when PSP was detected on PACXR in patients presenting with chest pain and respiratory distress. According to the findings of our study, it was thought that the probability of undergoing an invasive procedure and surgical intervention increased as the percentage of PTX detected in PACXR increased. Tube thoracostomy may be required in a patient with PSP if PTX does not start to decrease and lung expansion does not increase after an average of 60 hours after the decision for medical follow-up, and if PTX is progressive in the follow-up. VATS can be performed on a patient with PSP when lung expansion does not increase after an average of 18 hours after tube thoracostomy, when PTX progresses, when air leakage continues for more than 10 days despite increased lung expansion, and when recurrent PTX occurs.

## Introduction

Pneumothorax (PTX) refers to the accumulation of air in the pleural space, resulting in the collapse of the lung. Spontaneous PTX is a PTX that occurs without trauma or iatrogenic cause [1]. It is divided into primary and secondary PTX. Primary spontaneous PTX (PSP) occurs in healthy people without any underlying lung disease, whereas secondary spontaneous PTX develops in the presence of a specific disease in the lung [1]. The etiology of PSP is thought to be related to apical, subpleural bullae/blebs [2,3]. It is a rare disease in children under 18 years of age. In the pediatric population, the peak age of incidence is late adolescence, and it is more common in males [4]. PTX can cause symptoms such as chest pain, shortness of breath, chest tightness, cough, and sometimes tension PTX, which can be life threatening [5]. Generally, there are three approaches to the initial management of a patient with PSP: non-invasive medical monitoring, drainage by needle aspiration or chest tube placement, and surgery, usually video-assisted thoracoscopic surgery (VATS) or thoracotomy [6]. Some studies reported that nonsurgical treatment such as medical monitoring, aspiration, and tube thoracostomy will be sufficient for most patients with a first episode of PSP [7-9]. However, some studies have reported that recurrence will decrease with surgical treatment, VATS should be the standard approach in recurrent cases and should be considered for treatment even during the first episode [10]. Despite these recommendations, the ideal approach to the treatment of PSP remains controversial. All these controversies are based on the lack of pediatric specific guidelines for the treatment of PSP and the fact that it remains unclear whether adult guidelines are applicable. This study aims to review the PSP patients we have treated as well as the literature and to present our approach to the treatment of PSP in pediatric patients and VATS.

This article was previously posted to the Research Square preprint server on April 10, 2023.

## Materials and methods

The study was designed retrospectively and conducted at a tertiary referral university hospital between January 1, 2015, and May 1, 2021.

Eligibility criteria

Patients under the age of 18 years with a diagnosis of PTX were included in the study. Medical records were analyzed in terms of clinical characteristics, demographic data, findings from imaging data, procedures performed, and course of the disease at hospital. Recurrent PTX was defined as the development of a new ipsilateral PTX at any time following the treatment of the initial PTX. The development of contralateral PTX was considered as a separate and independent event. Thoracic computed tomography (TCT) scans were considered negative if there were no subpleural bullae/blebs in the lung. The PTX percentage = 100 − (collapsed area diameter (cm^3^)/hemithorax diameter (cm^3^)), a method specified by Light et al., was used to determine the PTX rate [11]. While the duration of lung expansion was recorded in hours by looking at the time of the radiographs taken, the durations of chest tube stay and hospitalization in patients who underwent tube thoracostomy were recorded in days. Patients were defined as under medical follow-up alone if they had not undergone any procedure or operation at first admission. Patients were defined as being treated with tube thoracostomy if a catheter or standard thoracostomy tube was placed during their hospitalization without any additional surgical intervention. Patients who underwent surgery (VATS) for the treatment of PSP during their hospitalization were defined as having undergone surgical treatment. In surgical treatment, mechanical pleurectomy was defined as partial pleurectomy if performed only at the apical level and total pleurectomy if performed on the entire parietal pleura.

Outcome parameters

Excluded from the study were patients with no evidence of PTX on radiologic imaging (postero-anterior chest X-ray (PACXR) or TCT), incomplete medical records for follow-up, history of trauma, and neonatal PTX.

Statistical analysis

IBM SPSS Statistics 22 software was used for statistical analyses while evaluating the findings obtained in the study. Kolmogorov-Smirnov and Shapiro-Wilks tests were used to evaluate the conformity of the parameters to normal distribution. In addition to descriptive statistical methods (mean, standard deviation, and frequency), in the comparison of quantitative data, one-way ANOVA was used for intergroup comparison of parameters that showed normal distribution, and the Tukey HDS test was used to determine the group causing the difference. The Kruskal-Wallis test was used for intergroup comparisons of parameters that did not show normal distribution, and Dunn's test was used to determine the group causing the difference. Student’s t-test was used for comparisons between two groups for normally distributed parameters and the Mann-Whitney U test was used for comparisons between two groups for non-normally distributed parameters. The chi-square test, Fisher's exact chi-square test, Fisher-Freeman-Halton exact test and continuity (Yates) correction were used to compare qualitative data. Pearson correlation analysis was used to examine the relationships between the parameters that conformed to normal distribution. Significance was evaluated at p < 0.05 level.

## Results

When the records were analyzed retrospectively, a total of 98 PTX cases were observed in 69 patients, 61 (88.4%) male and eight (11.6%) female.The ages of the patients ranged between 13 and 17 years with a mean of 16.59 ± 0.95 years. It was found that 48 (49%) PTX cases were treated with tube thoracostomy, 19 (19.4%) were treated with medical follow-up and 31 (31.6%) were treated with VATS. 50 (51%) PTX cases developed on the right side and 48 (49%) on the left side. The follow-up period varied between three and 78 months, with a mean follow-up period of 37.66 ± 19.57 months and a median of 36.5 months. It was found that all patients had chest pain and shortness of breath at the time of admission. It was observed that PACXR was applied to all PSP cases at the time of admission, and PTX was observed radiologically in all of them.TCT was performed on 60 of 69 patients (87%). The rate of presence of bullae/blebs on the same side with PTX on TCT was 48.3% (29 patients), and the rate of presence of contralateral bullae/blebs was 15% (nine patients).

Although the rate of recurrent PTX in patients with bulla/bubble on the ipsilateral side of PTX on TCT was found to be higher than that in patients without bulla/bubble on the same side of PTX, there was no significant difference between them (p > 0.05). There was no significant difference in the development of contralateral PTX in patients with and without contralateral bulla/bubble on TCT (p > 0.05) (Table 1).

**Table 1:** Evaluation of the rates of recurrent PTX and contralateral PTX in the follow-up of patients with bullae detected on CT Continuity (yates) correction and Fisher's exact test PTX: Pneumothorax; TCT: Thoracic computed tomography

PTX side	Same-side bullae with PTX on TCT		P value
	Yes (n = 29)	No (n = 31)	
	n (%)	n (%)	
Recurrent PTX at follow-up	10 (34.5%)	4 (12.9%)	0.095
	Bullae on the opposite side of PTX on TCT		
	Yes (n = 9)	No (n = 51)	
	n (%)	n (%)	
Contralateral PTX at follow-up	3 (33.3%)	3 (33.3%)	3 (33.3%)

When the PTX percentages at admission were analyzed according to treatment modalities in all PTX cases, the mean percentage of PTX was 62.51% in patients who underwent tube thoracostomy, 32.13% in patients who were followed-up medically, and 72.83% in patients who underwent VATS. There is a significant difference between these values (p: 0.000; p < 0.05). As a result of the Tukey HSD test performed to determine which treatment groups the significance originated from, the mean percentage of PTX in the lungs treated with VATS was found to be significantly higher than tube thoracostomy and medical monitoring methods (p1: 0.047; p2: 0.000; p<0.05). The mean percentage of PTX in patients treated with the tube thoracostomy method was significantly higher than that of the medical follow-up method (p = 0.000; p < 0.05) (Table 2).

**Table 2:** Evaluation of PTX percentage according to treatment methods One-way ANOVA Test *p < 0.05 PTX: Pneumothorax; VATS: Video-assisted thoracoscopic surgery

Treatment	PTX Percentage	p
	Ort ± SS	
Tube thoracostomy (n = 48)	62.51 ± 20.30	
Medical Monitoring (n = 19)	32.13 ± 16.63	0.000*
VATS (n = 31)	72.83 ± 16.84	

When the post-procedure re-expansion times were analyzed between the groups according to the treatment method for the initial PTX attack, re-expansion was observed as 18.3 hours on average in those who underwent tube thoracotomy, 60 hours on average in those who were treated with medical follow-up, and 31.4 hours on average in those who underwent VATS. There is a significant difference between these values (p: 0.000; p < 0.05). As a result of Dunn's test performed to determine from which groups the significance originated, the time to full expansion of the tube thoracostomy group was found to be significantly shorter than the medical monitoring and VATS groups (p1: 0.000; p2: 0.027; p < 0.05). There was no significant difference between the medical monitoring and VATS groups (p > 0.05) (Table 3).

**Table 3:** Evaluation of the time to re-expansion after the procedure between the groups according to the treatment method for the initial PTX attack Kruskal-Wallis test *p < 0.05 PTX: Pneumothorax; VATS: Video-assisted thoracoscopic surgery

Treatment	Time to re-expansion after the procedure (hours)
	Mean ± SD(median)
Tube thoracostomy	18.3 ± 14.6 (12)
Medical monitoring	60 ± 36.9 (48)
VATS	31.4 ± 20.5 (24)
p	0.000*

When the duration of chest tube stay according to the treatment method in the treatment of the initial PTX attack was analyzed, the mean duration of chest tube stay in the children treated with tube thoracostomy was 5.1 days (5.1 ± 2.5 (5)) (mean ± SD (median)), and the duration of chest tube stay in the children treated with VATS was 4.2 days (4.2 ± 1 (4)) (mean ± SD (median)). The mean duration of chest tube stay was found to be 5.44 days (5.44 ± 2.57 (5)) (mean ± SD (median)) in all PTXs treated with tube thoracostomy. In addition, when patients who underwent VATS because of prolonged air leak (PAL) were analyzed, the mean time from the time of tube thoracostomy until VATS was performed was 9.69 days. (9.69 ± 2.41 (10)) (mean ± SD (median)).

It was found that a total of 31 VATS procedures were applied to 28 patients. Follow-up time after VATS ranged from three to 78 months, with a mean of 31.5 ± 20.3 months and a median of 28 months. It was found that 18 (58.1%) VATS procedures were performed on the right side and 13 (41.9%) on the left side. It was determined that 9.7% of VATS procedures were performed due to previous contralateral PTX development, 38.7% due to recurrent PTX and 51.6% due to PAL. 58.1% of VATS procedures included partial pleurectomy and 41.9% total pleurectomy. In the surgical records, it was observed that bulla/bleb was detected in all patients during VATS (Table 4).

**Table 4:** VATS findings VATS: Video-assisted thoracoscopic surgery, PTX: Pneumothorax; PAL: Prolonged air leak

VATS Features	Description	n	%
Party	Right	18	58.1
	Left	13	41.9
Indication for VATS	Opposite side PTX	3	9.7
	Relapse	12	38.7
	PAL	16	51.6
Bullae/bleb during VATS	Yes	31	100
Pleurectomy	Partial	18	58.1
	Total	13	41.9

When the duration of chest tube stay after VATS was analyzed between those who underwent partial and total pleurectomy during VATS, it was found to be 5 days (4.56 ± 1.25 (5)) (mean ± SD (median)) in those who underwent partial pleurectomy and 4 days 4.69 ± 1.44 (4) (mean ± SD (median)) in those who underwent total pleurectomy. No significant difference was observed between these values (p: 1,000, p > 0.05, Mann-Whitney U Test). When the time to full expansion after VATS was analyzed between those who underwent partial and total pleurectomy during VATS, it was found to be 24 hours (27.5 ± 18.07 (24)) (mean ± SD (median)) in those who underwent partial pleurectomy and 24 hours (36.0 ± 22.98 (24)) (mean ± SD (median)) in those who underwent total pleurectomy. No significant difference was observed between them (p: 0,300, p > 0.05, Mann-Whitney U Test).

## Discussion

​​​​​​Although the incidence of PSP in the general population is 6-18 per 100,000, some publications report that the incidence in children is 0.1% to 3-4 per 100,000. Some researchers have observed that PSP occurs predominantly in males, with a male-to-female ratio ranging from 2:1 to 9:1 [12,13]. In pediatric studies, the highest incidence occurs between 14 and 17 years of age, mainly in late adolescence [14]. In our study, PSP was more common in males with a ratio of 8:1, and the mean age was approximately 16 years, consistent with the literature. In addition, in our study, 51% of PTX cases were observed on the right side and 49% on the left side.

Kuo et al. reported that PSP occurred most frequently at rest, with acute chest pain, shortness of breath, and chest tightness as the most common clinical symptoms [4]. Similarly, chest pain and shortness of breath were the most common symptoms in our study. PACXR is diagnostic when PSP is suspected in patients with these symptoms [15]. In our study, it was observed that all patients underwent PACXR for the diagnosis of PSP. Although PSP is diagnosed with PACXR, it is obvious that TCT scanning is much more sensitive than PACXR in terms of detection of bullae/blebs and parenchymal lesions [16]. However, the role of TCT in the management of PSP patients remains controversial. The presence, size, and location of bullae/blebs can be determined by TCT scan at the patient's first PSP attack. However, TCT findings may not always coincide with intraoperative findings. In a study by Lopez et al., although bullae/blebs were detected in only 60% of PSP patients who underwent TCT, bullae/blebs were detected intraoperatively in 98% of patients who underwent surgery [13]. Similarly, in our study, although bullae/blebs were detected in 48.3% of our patients who underwent tomography, bullae/blebs were detected in all patients who underwent VATS. Some publications have reported a higher risk of recurrence in patients with bullae/blebs detected on TCT scan in the first PTX attack [16,17]. On the contrary, Martinez-Ramos et al. reported no correlation between the presence or absence of bullae/blebs on TCT scans and PSP recurrence [18]. Some studies have also reported that the presence of bullae/blebs on TCT should not affect the surgical decision and decision making in preventing recurrence [12,18]. Lopez et al. reported that there is no evidence to support prophylactic VATS in asymptomatic patients with bullae/blebs detected during TCT scanning [13]. In our study, no significant difference was observed between patients with and without ipsilateral bullae/blebs detected on TCT at the first PSP attack in terms of the development of recurrent PTX. Likewise, no significant difference was observed in terms of development of PTX in the follow-up of patients with and without contralateral bullae/blebs detected on TCT at the first PSP attack. These results suggested that detection of ipsilateral or incidental contralateral bulla/bubble on TCT may not be predictive for the follow-up of ipsilateral recurrent PTX or contralateral PTX.Thus, we think that TCT scanning in the first PSP attack would not be beneficial in terms of both PTX follow-up and surgical decision. However, like O'Lone et al., we think that TCT scanning can be performed for recurrent PSP to evaluate the possible pathological structure of the lung and to guide surgical treatment [19].

There are several treatment options for the first episode of PSP, including observation, needle aspiration, tube thoracostomy, and surgery (thoracotomy, VATS). The American College of Chest Physicians (ACCP) recommends observation in the emergency department in clinically stable patients with small PTX, whereas those with large PTX should be hospitalized with chest tube or pigtail catheter placement [20]. According to the British Thoracic Society 2010 guidelines, observation is the preferred treatment for PSP less than 15% without significant dyspnea, and up to 80% of these have no persistent air leak [21]. In progressive PTX and PTX above 15%, aspiration with a catheter or tube thoracostomy has been recommended [12,21,22].

In our study, when evaluated according to treatment methods, the mean PTX percentage was found to be 72% in those treated with VATS, 62% in those treated with tube thoracostomy, and 32% in those treated with medical follow-up. These results suggested that invasive procedures are inevitable as the percentage of PTX increases. In addition, our study made us think that the medical follow-up recommended for PTX up to 15% in other studies can be applied up to 32% in PTX according to the results of our study. In addition, it was seen that tube thoracostomy may be required for those with a PTX percentage above 32%, and VATS for those with a PTX percentage above 72%.

Successful treatment of PTX patients is defined as positive lung re-expansion seen on PACXR [23]. In our study, it was found that lung re-expansion was achieved in approximately 60 hours in patients with PTX who underwent medical follow-up. If there is no shrinkage in the PTX and no increase in lung expansion after this period, we thought that tube thoracostomy may be performed. However, if PTX is progressive during medical follow-up, it is recommended that an interventional procedure (thoracentesis, tube) should be performed without waiting [12,22]. Furthermore, Sahn et al. reported that supplemental oxygen support during medical monitoring may accelerate the reabsorption of air [12]. In contrast, Fry et al. reported that supplemental oxygen support may facilitate the reaccumulation of air in the pleural space up to four times faster [24]. The diversity of opinions on this issue reveals different views on the application of supplemental oxygen therapy. In our study, it was observed that additional oxygen support was given to patients with clinical findings such as dyspnea and low O_2_ saturation.

In the treatment of pediatric PSP, there is no consensus on how many days after the tube thoracostomy application, the persistence of air leakage should be considered as PAL, and surgery (VATS or thoracotomy) should be performed. Although ACCP recommends surgery for patients treated with tube thoracostomy with air leakage lasting longer than 4 days, some publications recommend a period ranging from three to seven days [3,24-26]. In our study, in patients who underwent tube thoracostomy in the treatment of the first attack, the mean time to lung reexpansion was 18 hours, and the mean time to stay in the chest tube was five days.This result suggests that in patients who underwent tube thoracostomy in the first attack, those who did not achieve lung re-expansion after 18 hours and needed tube thoracostomy for more than five days are candidates for surgery (VATS or thoracotomy). In addition, when we analyzed the patients who underwent VATS for PAL in our study, we found that the mean time from the time of tube thoracostomy until VATS was performed was 10 days. In cases treated with tube thoracostomy both in the first attack and in total PTX, the mean duration of chest tube stay, in other words, recovery time, was found to be five days. With these results, we thought that physicians could wait at least five days to evaluate the patient as a PAL. Therefore, it should be considered that PAL may develop in air leaks lasting longer than five days, and we thought that air leaks lasting longer than 10 days can be considered as PAL and surgery (VATS or thoracotomy) can be performed.

Because there are no adequate guidelines for the treatment of PSP in pediatric patients, the treatment is generally based on adult data, but it remains unclear whether these adult guidelines are valid for children. ACCP recommends surgery for adult patients with PALs and recurrent PSP [20]. Some studies also recommend surgical treatment of PSP in patients with recurrent ipsilateral or contralateral PTX and in patients with PAL [25,27]. In our study, 9.7% of the indications for VATS were development of contralateral PTX, 38.7% were recurrent PTX, and 51.6% were PAL. According to our results, the most important factors for the decision for surgery (VATS or thoracotomy) appear to be PAL and recurrent PTX. However, there is a broad consensus among pediatric surgeons to use a minimally invasive approach (98%) and to perform stapler blebectomy (97%) and mechanical pleurodesis (77%) when surgical intervention is decided [27]. With its diagnostic and therapeutic advantages, VATS is a generally accepted technique. Its advantages over thoracotomy include better visualization of the thoracic cavity, reduced post-operative pain and morbidity, and earlier recovery [25]. In our study, it was observed that VATS was applied to all patients for whom surgery was decided. The goal in the treatment of PSP is to achieve lung re-expansion and prevent recurrence with minimal morbidity [28]. Although some studies have reported the advantages of VATS over thoracotomy, others have suggested that VATS may still be associated with a slightly higher recurrence rate [10,20,21,29]. Pleural abrasion and pleurectomy procedures can be performed with VATS to reduce recurrence rates. However, the guidelines do not clearly define the surgical procedure [10,20]. Although these procedures reduce the recurrence rate, they may cause local adhesions and development of severe hematoma, especially after pleurectomy [30]. Total pleurectomy may increase these complications. In our study, it was found that there was no significant difference between total and partial pleurectomy in terms of the time to complete lung re-expansion and the duration of chest tube stay after VATS. In terms of recurrence, no recurrence was observed in 13 patients who underwent total pleurectomy, whereas recurrence was observed in only one of 18 patients who underwent partial pleurectomy. With these findings, we think that partial pleurectomy is more advantageous during VATS. Thus, we believe that complications of pleurectomy such as hematoma and bleeding can be minimized.

Limitations

Our retrospective study needs to be supported by prospective studies. Additionally, another one of its limitations is the fact that the study was conducted in a single center. 

## Conclusions

Our retrospective study showed that TCT scanning did not provide additional benefit when PSP was detected on PACXR in patients presenting with chest pain and respiratory distress. According to the findings of our study, it was thought that the probability of undergoing an invasive procedure and surgical intervention increased as the percentage of PTX detected in PACXR increased. Tube thoracostomy may be required in a patient with PSP if PTX does not start to decrease and lung expansion does not increase after an average of 60 hours after the decision for medical follow-up, and if PTX is progressive in the follow-up. VATS can be performed on a patient with PSP when lung expansion does not increase after an average of 18 hours after tube thoracostomy, when PTX progresses, when air leakage continues for more than 10 days despite increased lung expansion, and when recurrent PTX occurs. In addition, partial pleurectomy seems to be more advantageous during VATS.
